# Integrate analysis of the promote function of Cell division cycle-associated protein family to pancreatic adenocarcinoma

**DOI:** 10.7150/ijms.53243

**Published:** 2021-01-01

**Authors:** Chen Xing, Zhenglin Wang, Yating Zhu, Chao Zhang, Miao Liu, Xianyu Hu, Wei Chen, Yinan Du

**Affiliations:** 1 School of Basic Medical Sciences, Anhui Medical University, 230032 Hefei, Anhui, China; 2 Department of General Surgery, The First Affiliated Hospital of Anhui Medical University, 230022 Hefei, Anhui, China

## Abstract

**Background:** The cell division cycle-associated (CDCA) protein family plays a pivotal role in the regulation of the cell cycle during tumorigenesis and predicts the prognosis of tumors, but an analysis of these proteins in pancreatic adenocarcinoma (PAAD) is still lacking.

**Methods:** Oncomine and GEPIA were used to observe the expression and prognostic value of eight CDCAs in pan-cancer. Univariate Cox analysis of single CDCAs and multivariate Cox analysis of all eight CDCAs were performed to evaluate the integrated prognostic value of CDCAs, and the results are displayed as hazard ratios (HRs) and 95% confidence intervals (95% CIs). K-M plots and receiver operating characteristics curves were used to display the predicted function and accuracy of CDCAs to determine the risk score. Annotation of CDCA-related genes, gene sets enrichment analysis (GSEA) and gene sets variation analysis (GSVA) were performed to reveal the CDCAs that impact biological processes.

**Results:** CDCAs expression in most tumors is higher than that in normal tissues and is associated with a poor prognosis. Regarding PAAD, increased CDCA expression along with advanced PAAD tumor stage, NUF2, CDCA2, CDCA3, CDCA4 and CDCA5 expression are risk factors for poor prognosis, while CBX2 expression is a protective factor (P < 0.05). The integrated prognostic value of CDCAs in PAAD patients was validated by SurvExpress in the TCGA-PAAD cohort (P < 0.001, HR = 2.16, 95% CI = 1.41-3.3) and the ICGC-PACA cohort (P < 0.001, HR = 2.56, 95% CI = 1.73-3.79). Genetic alteration and DNA methylation of CDCAs might not affect the prognosis of PAAD patients. After comparing high- and low-risk groups separated by CDCA risk scores, the activated pathways were revealed and included the cell cycle, DNA repair, P53, MYC-targets, E2F-targets and PI3K pathways.

**Conclusion:** CDCAs can predict the OS prognosis of PAAD patients. The cell cycle, DNA repair, E2F, P53 and PI3K signaling pathways, in which CDCAs are involved, impact the tumorigenesis of PAAD.

## Introduction

Pancreatic cancer is an increasingly common tumor worldwide, and approximately 85% of cases are consistent with pancreatic adenocarcinoma (PAAD)^1^. PAAD is fourth most frequent cancer and was the seventh most common cause of cancer-related death in 2018 worldwide, accounting for approximately four hundred thirty thousand deaths^2^. Increasing age is a risk factor for the incidence and mortality of PAAD, while PAAD is more frequent in males than in females^2^. Smoking cigarettes, obesity, heavy alcohol abuse and family history are known risk factors for PAAD patients^3^-^5^. Smoking cigarettes can alter the microenvironment of pancreatic tissue, lead to long-standing inflammation, increase onco-miRNA expression, induce KRAS mutations and affect enzyme secretion^6^. Asahina et al. reported that even moderate alcohol use could induce an advanced stage of PAAD in Kras^G12D^ mutant mice^7^. The median overall survival (OS) time for advanced stage PAAD is less than 1 year, while the 5-year survival for patients with all stages of PAAD is less than 10%^8^, ^9^. Therefore, it is necessary to identify prognostic biomarkers for PAAD patients to guide clinical treatment.

An important strategy for tumor therapy focuses on the inhibition of tumor cell proliferation, which is regulated by the three major checkpoints in the cell cycle: G1, G2/M transition and metaphase-to-anaphase transition^10^. The cell division cycle-associated (CDCA) protein family is a gene set that is deeply involved in the process of the cell cycle and contains eight homologous proteins: CDCA1 is also known as NUF2 Component of NDC80 Kinetochore Complex (NUF2), and CDCA6 is chromobox 2 (CBX2). CDCA2 can control the protein phosphatase 1 (PP1)γ-dependent DNA damage response and promotes major mitotic histone H3 dephosphorylation in a PP1-dependent manner^11^, ^12^. CDCA3 contains an F-box motif and participates in the G2/M phase of the cell cycle, which can promote cell proliferation through the NF-κB/cyclin D1 and E2F1/p21 pathways^13^, ^14^. CDCA5 is recognized as the substrate of the anaphase-promoting complex, which is essential for the stability of cohesion and chromatid binding at the S and G2/M phases and is then degraded in a ubiquitin-dependent manner in the G0/G1 phase^15^, ^16^.

Several studies have illustrated the prognostic value of CDCAs in tumors. Meng et al.^17^ reported the increased risk associated with NUF2, CDCA3, CDCA4, CDCA5, CDCA7, and CDCA8 expression in renal cell carcinoma and the decreased risk associated with CDCA2 expression. Zhang et al.^18^ found the risk associated with NUF2, CDCA2-5, and CDCA8 expression in endometrial carcinoma, while CDCA7 was a protective factor. However, the prognostic value of CDCAs in PAAD has not been demonstrated. Therefore, in the current study, we elucidated the association between eight CDCAs and the OS of PAAD patients, as well as the integrative CDCA prognostic signature. The potential implications of the genetic alteration and DNA methylation of CDCAs for prognosis were also considered, and the potential signaling pathways impacted by CDCAs were assessed.

## Methods

### Pan-cancer analysis for the predicted value of CDCAs

To globally understand the function of CDCAs, we used the Oncomine publicly online cancer microarray database^19^. This database can display comparable mRNA expression profiles of normal and tumor tissues in different types of cancers obtained from diverse cohorts. The comparation between normal and tumor tissues was analyzed by Student's t-test, with the following thresholds: P value < 0.05; fold change > 1.5; gene rank, top 10%; data type, all. The pan-cancer prognostic values of eight CDCAs were also evaluated by the GEPIA^20^. The hazard ratio (HR) of each gene to the overall survival (OS) in different tumors was calculated by univariate Cox regression analysis.

### Prognostic value of CDCAs in PAAD patients

The comparison of PAAD tumor tissue and normal pancreatic tissue was performed by GEPIA. The mRNA expression levels of eight CDCAs were extracted from 179 PAAD tumor tissues in the TCGA-PAAD cohort and 171 normal pancreatic tissues from the TCGA-PAAD cohort and the GTEx dataset. All the mRNA expression values were pre-normalized by log_2_(TPM+1). The mRNA expression data of eight CDCAs distributed in different tumor stages and grades were downloaded from the ULCAN^21^. The original gene expression files of eight CDCAs and their clinical features were downloaded from the UCSC Xena (https://gdc.xenahubs.net/download/TCGA-PAAD.htseq_fpkm.tsv.gz). The transcripts per million (TPM) read data were calculated from the fragments per kilobase of non-overlapped exons per million fragments (FPKM) value and were then modified to the form of log_2_(TPM+1). A heatmap illustrated by the pheatmap R package was constructed to display the association and distribution between CDCAs and clinical features. K-M survival was used to show the diverse OS outcomes in patients with high or low expression of CDCAs, which are separated by the median value of each gene expression level. The P value to show the difference in OS outcome in K-M survival was calculated by log-rank test, while the HR and 95% CI were obtained by Cox regression analysis in two groups. To investigate the integrative prognostic value of CDCAs, we used a public resource-based survival assessment platform, SurvExpress^22^ with the TCGA-PAAD cohort and the ICGC-PACA cohort. The K-M plot and receiver operating characteristic (ROC) curve were displayed with the combined values of the eight CDCAs.

### Genetic alteration and DNA methylation effects on prognosis

Genetic alterations, including gene mutations and copy number alterations, are the potential factors impacting expression. We evaluated the genetic alterations of CDCAs with Oncoprinter from cBioportal and the impacts of CDCAs on PAAD patient survival^23^, ^24^. DNA methylation is another risk factor that affects the expression of CDCAs. The influence of DNA methylation on CDCA expression was assessed by DNMIVE^25^, and the impact of a single-methylation CpG site on the OS of PAAD patients was analyzed by MethSurv^26^.

### CDCAs impact on signaling pathways

To evaluate the impact of CDCAs on signaling pathways, we first identified the coexpressed genes of eight CDCAs from ULCAN^21^. The thresholds were set as R higher or equal to 0.3 and P value less than 0.05. The genes that met the thresholds for all eight CDCAs were defined as the CDCA-impacted genes (CIGs). Then, we annotated the enrichment of these genes by Metascape^27^ to reveal the potential mechanisms regulated by CDCAs. With GSEA analysis of KEGG pathways, we also highlighted the significantly different biological pathways in PAAD patients with CDCAs to determine the high- and low-risk groups^28^. The enrichment score (ES) was summed from the genes from a certain gene set if they met the top genes in all the ranked gene lists and was subtracted if the genes met the bottom genes. Normalized ES (NES) is used to adjust the duplicated analysis among different gene sets. Furthermore, gene-set variation analysis (GSVA) was also employed to assess the activated signaling, which could calculate samplewise gene-set enrichment with a Kolmogorov-Smirnov-like rank statistic; however, genes are often ranked using a kernel estimation of a cumulative density function. We performed GSVA analysis to evaluate the 50 HALLMARK gene signatures.

### Statistics

K-M survival analysis was used to indicate the different OS level of the high and low groups with the 'survminer' package, R version 3.6.5. Univariate Cox regression analysis was employed to calculate the hazard ratio (HR) and 95% confidence interval (95% CI). Comparisons of continuous data between two groups were performed with the Student's T-test. A two-sided P value less than 0.05 was considered statistically significant.

## Results

### CDCAs expression increased in various cancers and was associated with poor prognosis

**Figure 1:**
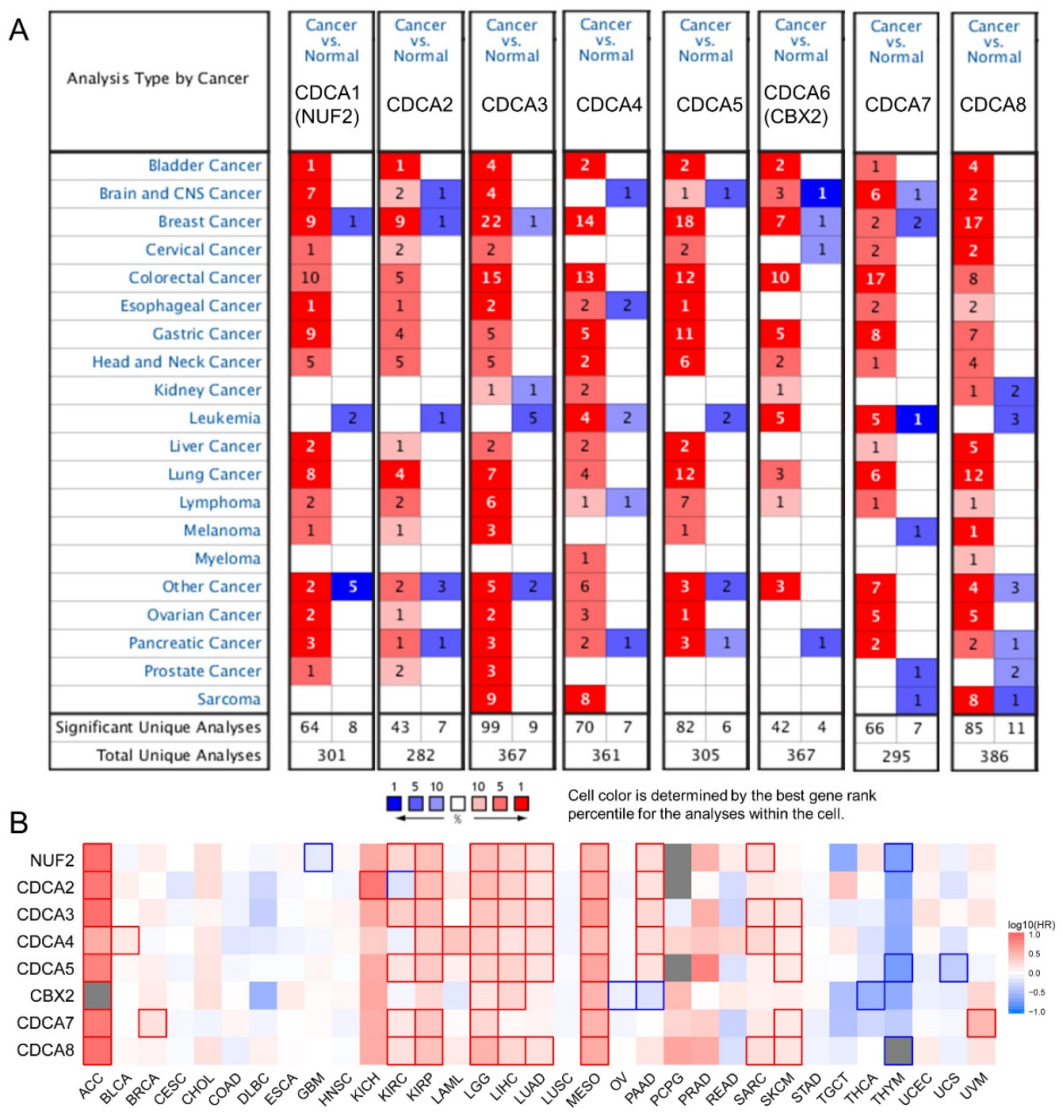
** The association between CDCAs with pan-cancer.** (A) CDCAs expression in tumor and normal tissues in pan-cancer evaluated by Oncomine; (B) CDCAs correlated with the overall survival of pan-cancer. The red and blue frame indicate the *P* value less than 0.05.

We first used Oncomine to globally understand the expression of CDCAs in tumor and normal tissues. We revealed that the eight CDCAs were increased in most tumors, but not leukemia and myeloma (**Figure 1A**). Furthermore, with the help of GEPIA, we comprehensively evaluated the prognostic value of CDCAs for OS. As shown in **Figure 1B**, red indicated increased risk, blue indicated decreased risk, and the bold border indicated a P value less than 0.05. We revealed that the eight CDCAs acted as risk factors for tumorigenesis in most tumors but as protectors in thymoma and thyroid cancer. In PAAD, NUF2, CDCA2, CDCA3, CDCA4 and CDCA5 are risk factors for poor prognosis, while CBX2 is a protective factor (P < 0.05).

### CDCAs were increased in advanced PAAD

**Figure 2:**
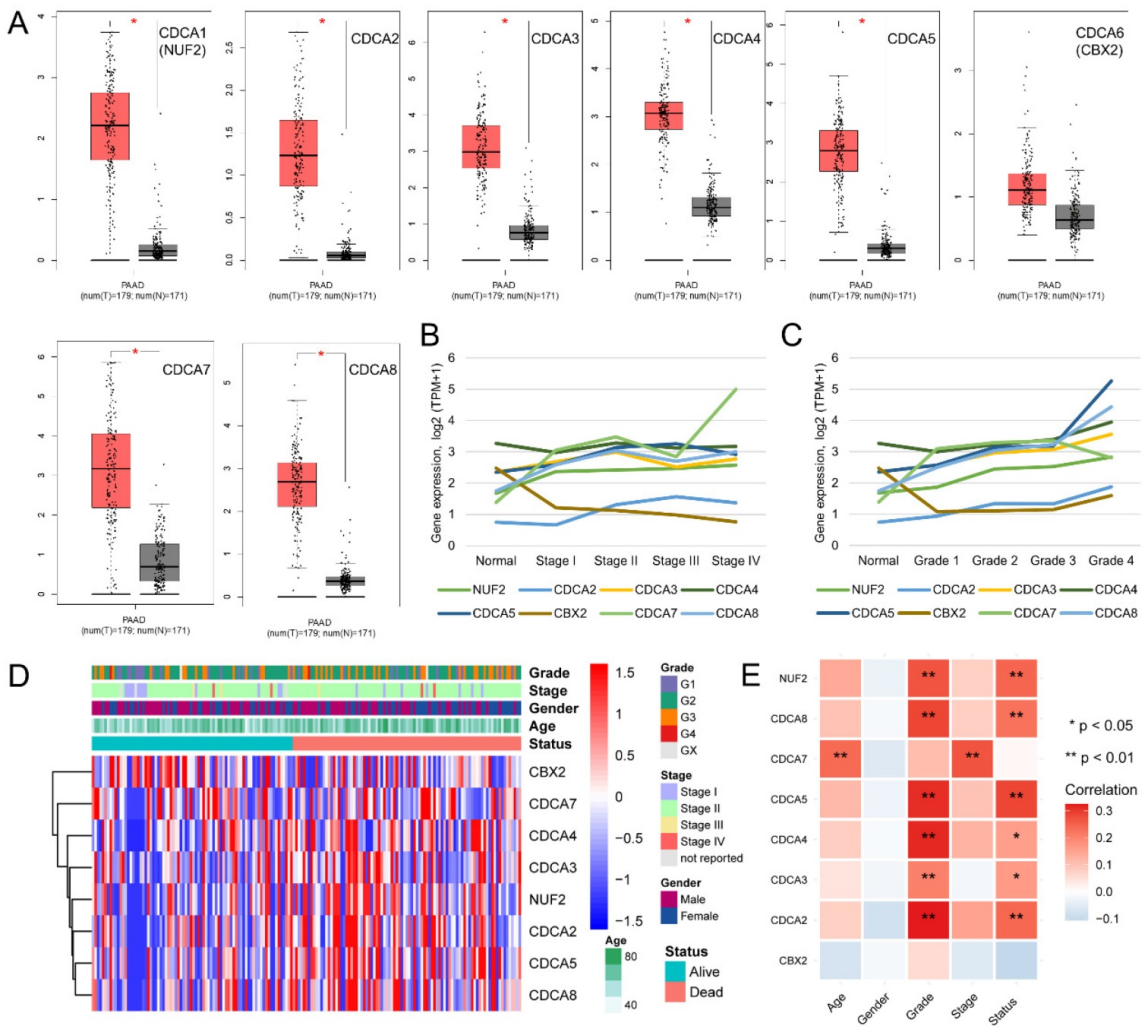
** The association between CDCAs with PAAD.** (A) CDCAs increased in tumor tissues as compare to normal tissues; (B) The mRNA level of CDCAs elevated along with the increased tumor stage; (C) The mRNA level of CDCAs elevated along with the increased tumor grade; (D) Heatmap to show the distribution of CDCAs and clinical features of PAAD patients. (E) Correlation between CDCAs and clinical features. *, *P* < 0.05

We evaluated the expression levels of eight CDCAs in GEPIA, which contains 179 PAAD tumor tissues from a TCGA-PAAD cohort and 171 normal pancreatic tissues from the TCGA-PAAD cohort and the GTEx database. We found that the expression levels of seven of eight CDCAs were increased in tumor tissues (P < 0.05), while there was no significant difference of CBX2 expression between tumor and normal tissues (**Figure 2A**). Furthermore, we noted the tendency that the expression of eight CDCAs increased with advancement of the PAAD tumor stage, especially for tumor grade (**Figure 2B-C**). The distribution of the expression levels of eight CDCAs and the clinical features of the PAAD patients from the TCGA-PAAD cohort are shown in **Figure 2D**. We revealed that most CDCAs positively associated with the advanced tumor stage and grade, as well as the outcome of dead (**Figure 2E**).

### CDCAs are associated with the prognosis of PAAD patients

**Figure 3:**
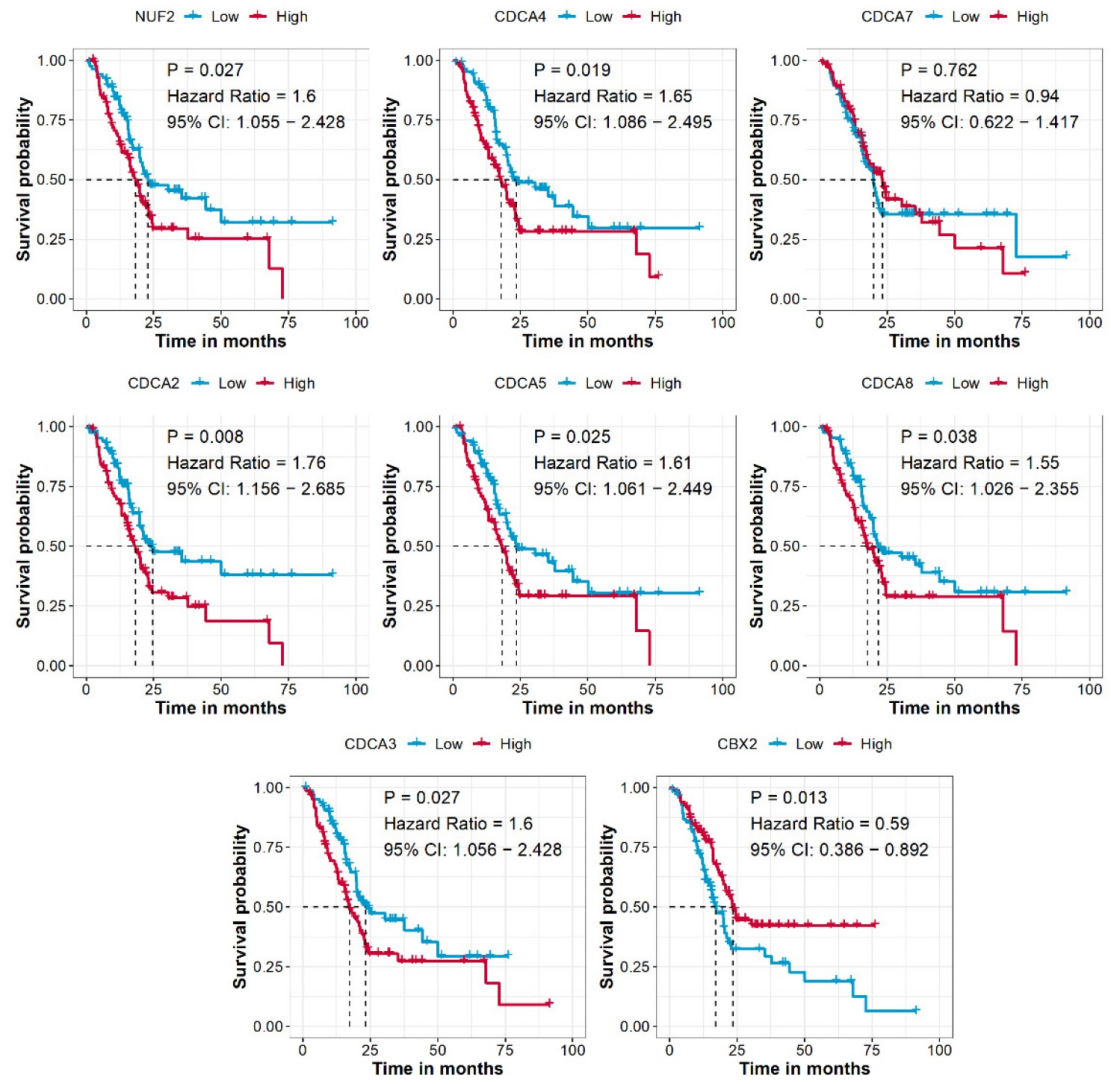
** CDCAs associated with the prognosis of PAAD patients.** Cut-off value: median expression of CDCAs.

After combining the mRNA expression data and clinical information, a total of 176 PAAD patients from the TCGA-PAAD cohort were used to analyze the prognostic prediction value of CDCAs. We revealed that higher expression levels of NUF2 (P = 0.027, HR = 1.6, 95% CI = 1.055-2.428), CDCA2 (P = 0.008, HR = 1.76, 95% CI = 1.156-2.685), CDCA3 (P = 0.027, HR = 1.6, 95% CI = 1.056-2.428), CDCA4 (P = 0.019, HR = 1.65, 95% CI = 1.086-2.495), CDCA5 (P = 0.025, HR = 1.61, 95% CI = 1.061-2.449), and CDCA8 (P = 0.038, HR = 1.55, 95% CI = 1.026-2.355) indicated an unfavorable OS for patients compared to genes with lower expression levels, while higher expression of CBX2 (P = 0.013, HR = 0.59, 95% CI = 0.386-0.892) was associated with a favorable prognosis (**Figure 3**).

### Integrated prognostic value of CDCAs in PAAD patients

**Figure 4:**
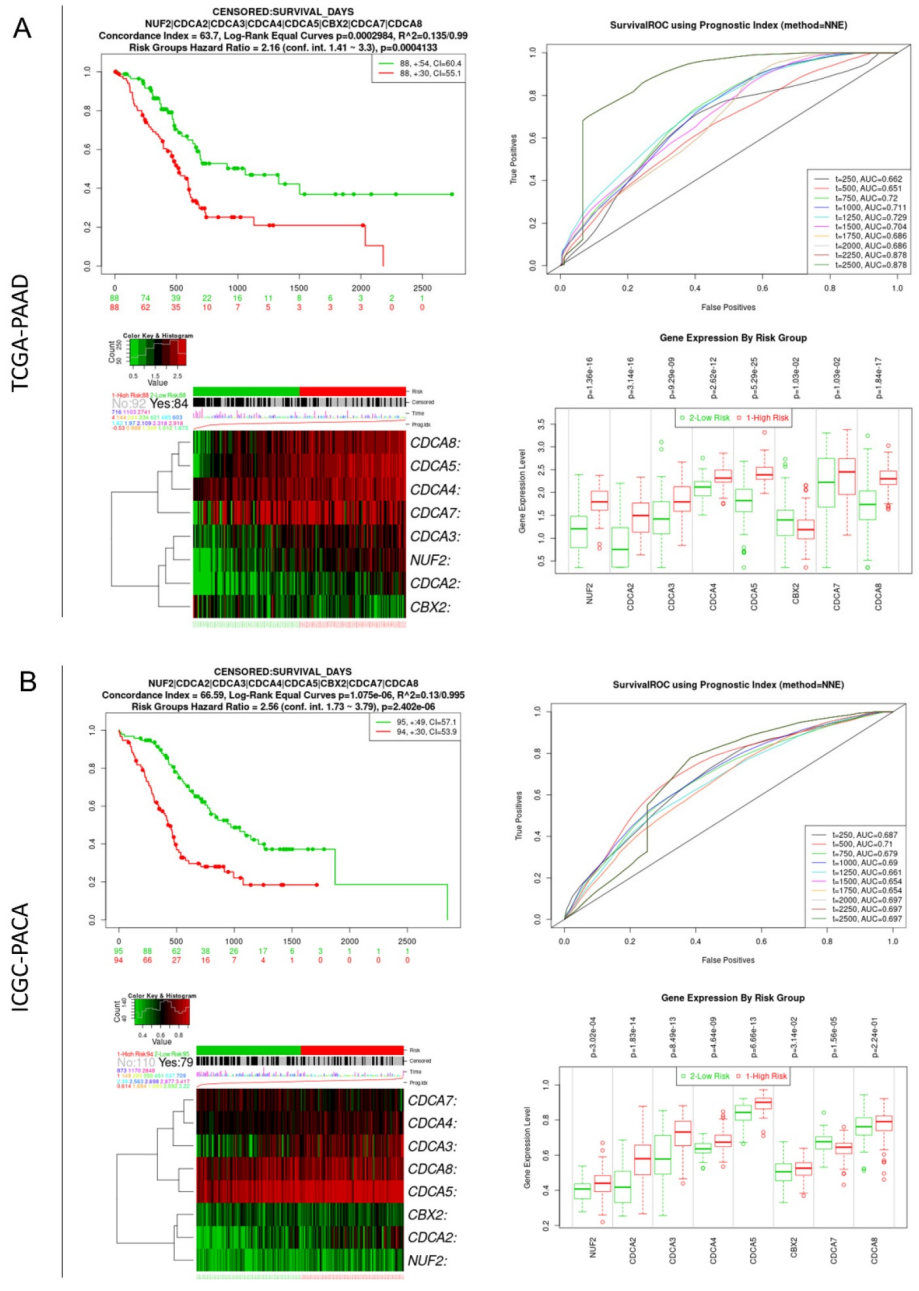
** Integrate prognostic value of CDCAs in TCGA-PAAD and ICGC-PACA cohorts.** (A) TCGA-PAAD cohort: K-M plot and ROC curve showing the integrate prognostic value of CDCAs, heatmap showing the different distribution of CDCAs expression, bar plot illustrating the mRNA level of CDCAs in high- and low-risk groups; (B) ICGC-PACA cohort: K-M plot and ROC curve showing the integrate prognostic value of CDCAs, heatmap showing the different distribution of CDCAs expression, bar plot illustrating the mRNA level of CDCAs in high- and low-risk groups.

The SurvExpress online platform was used to evaluate the integrative prognostic value of the eight CDCAs. For the TCGA-PAAD cohort, the risk score for patients was calculated based on the coefficients of CDCAs, which were generated by multivariate Cox regression analysis. Patients with high risk scores showed poorer OS than those with low risk scores (P < 0.001, HR = 2.16, 95% CI = 1.41-3.3). The time-dependent ROC curve showed that the predictive accuracy of CDCAs ranged from 0.662 to 0.878. Increased expression levels of NUF2, CDCA2, CDCA3, CDCA4, CDCA5, CDCA7, and CDCA8 were observed in the high-risk group, while decreased expression of CBX2 was observed in the high-risk group (**Figure 4A**). We also used the ICGC-PACA cohort to validate the prognostic value of CDCAs. The patients in the high-risk group also had a poor prognosis (P < 0.001, HR = 2.56, 95% CI = 1.73-3.79). The time-dependent ROC curve revealed that the predictive accuracy of CDCAs ranged from 0.687 to 0.710. Increased expression levels of NUF2, CDCA2, CDCA3, CDCA4, CDCA5, and CDCA8 were observed in the high-risk group of the ICGC-PACA cohort (**Figure 4B**).

### DNA methylation might indicate a diverse prognosis, but not genetic alterations

**Figure 5:**
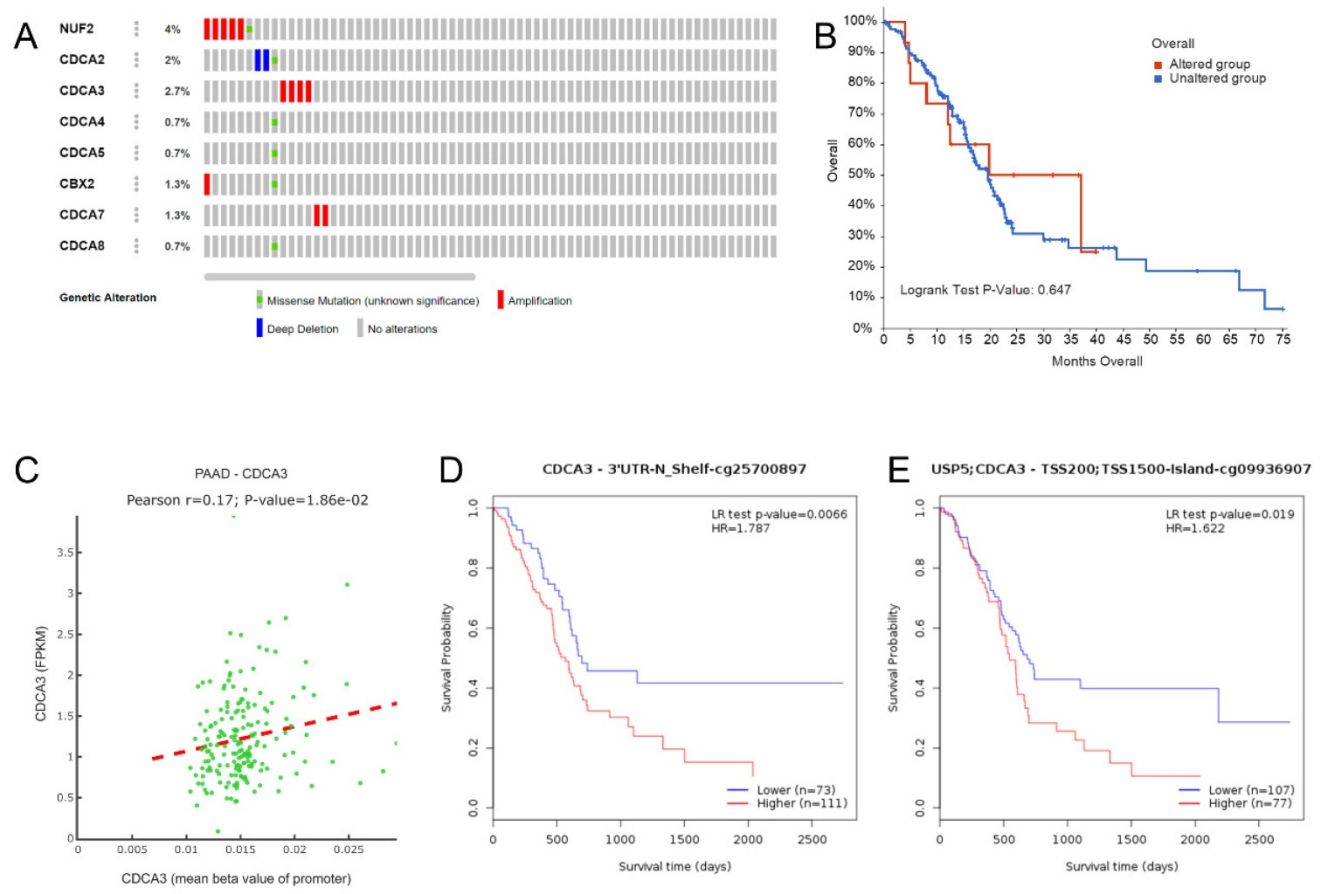
** CDCAs genetic alteration and DNA methylation effects to PAAD.** (A) Distribution of genetic alteration of eight CDCAs; (B) CDCAs genetic alteration did not impact the OS of PAAD patients; (C) The DNA methylation of CDCA3 impacted to its expression; (D) CDCA3-cg25700897 methylation promoted the poor prognosis of PAAD patients; (E) CDCA3-cg09936970 methylation promoted the poor prognosis of PAAD patients;

We obtained the genetic alteration data of CDCAs in the TCGA-PAAD cohort from cBioportal. NUF2 had the highest frequency of genetic alteration (4%), while only 0.7% of patients had genetic alterations in CDCA4, CDCA5, and CDCA8 (**Figure 5A**). Patients with genetic alterations in the CDCAs did not show different OS rates compared with those without the alterations (**Figure 5B**). Regarding DNA methylation, we first evaluated the promoter methylation and gene expression levels and only found that the promoter methylation of CDCA3 was positively associated with mRNA expression (**Figure 5C**). Additionally, we revealed the impact of single CpG to PAAD prognosis, which is displayed in Table 1. In particular, the increased methylation β values of the CDCA3-3'UTR-N shelf-cg25700879 site (P = 0.007, HR = 1.787) and the CDCA3-TSS200/TSS1500-island-cg09936970 site (P = 0.019, HR = 1.622) reflected a worse OS (**Figure 5D-E**).

### CDCAs were involved in cell cycle, DNA replication and DNA repair

**Figure 6:**
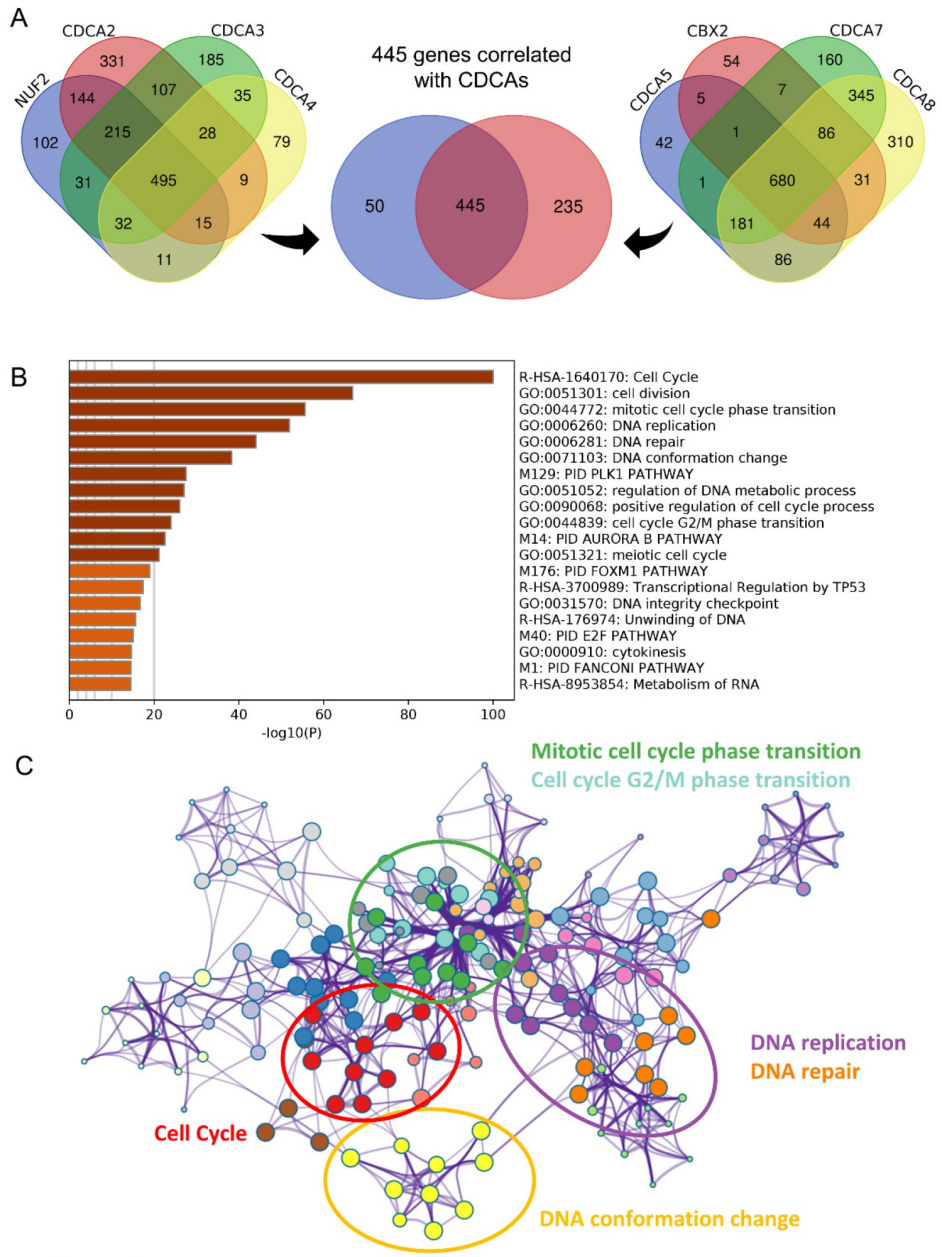
** Biological processes implicated by CDCAs** (A) The 445 correlated genes to eight CDCAs; (B-C) The annotation of biological processes based on the 445 genes.

**Figure 7:**
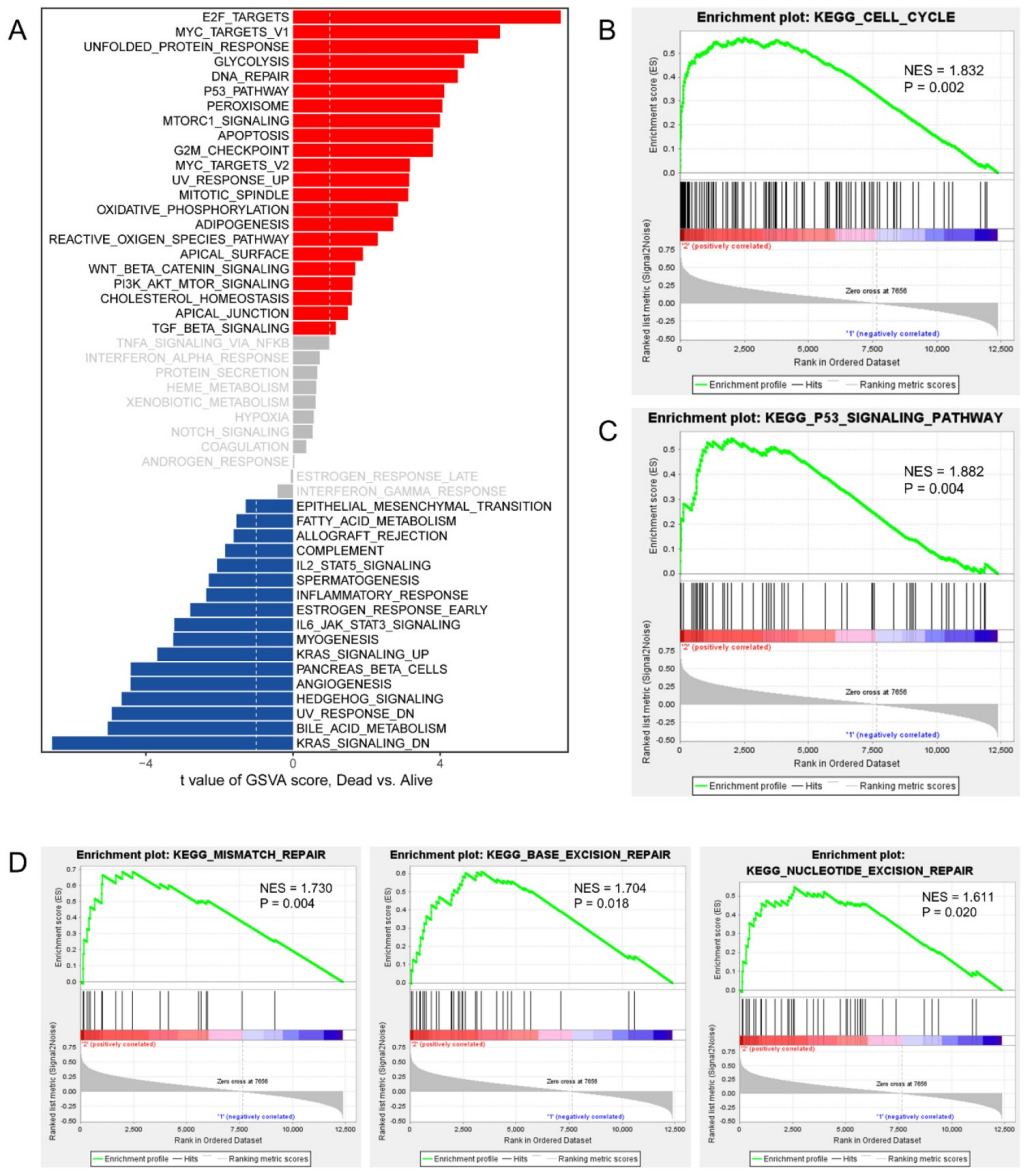
** Activated signaling pathways in CDCAs determined high-risk PPAD patients.** (A) GSVA analysis showing the activated hallmark pathways; Cell cycle pathway (B), P53 signaling pathway (C) and DNA repair pathways (D) activated in CDCAs determined high-risk PPAD patients.

To invastigate the mechanism of action of CDCAs, we used different methods. First, we merged the genes with correlations with CDCAs higher or equal to 0.3 based on Pearson analysis and P values less than 0.05. A total of 445 genes were associated with the eight CDCAs (**Figure 6A**). The 445 genes were enriched in key biological processes, including cell cycle, cell cycle G2/M phase transition, DNA conformation change, DNA replication and DNA repair (**Figure 6B-C**). Moreover, we compared the activated signaling pathways of the 50 key Hallmark cancer pathways and found that E2F-targets, MYC-targets, P53 pathway, and PI3K signaling were activated in the CDCA-delineated high-risk group and were associated with the DNA repair and G2M checkpoint pathways (**Figure 7A**). Additionally, similar results were also observed in the GSEA analysis. Activated KEGG cell cycle (**Figure 7B**), the KEGG P53 signaling pathway (**Figure 7C**), and DNA repair-associated pathways were observed in the CDCA-delineated high-risk group (**Figure 7D, Table 2**).

## Discussion

PAAD is one of the most dangerous tumors and is highly challenging to diagnose in the early stage^29^; thus, most PAADs are well-advanced at the time of diagnosis, while only 7% of PAADs are at the localized stage at the time of diagnosis^30^. The 5-year survival rates remain as low as 3% to 15%^31^. In addition, it is predicted that PAAD will be the second most frequent cause of cancer-related deaths in the United States in 2030^32^. Several factors can increase the risks of PAAD in patients, including pancreatic cystic lesions, familial inherited risk, and type 2 diabetes diagnosed at an age older than 50 years^33^, ^34^. In addition, it is important to define the prognostic markers for PAAD to guide clinical treatment. Chung et al.^35^ reported the prognostic value of serum fibrinogen to PAAD patients; serum fibrinogen expression was significantly higher in patients with distant metastasis, and the median OS was longer in patients with lower serum fibrinogen levels. Wu et al.^36^ identified three immune-related genes (CKLF, ERAP2, and EREG) and determined the prognostic signature of PAAD patients. Patients with high-risk scores were associated with a poor prognosis, with AUC values of 0.612 to 0.687. Moreover, Suenage et al.^37^ used peritoneal lavage tumor DNA (ptDNA) to predict the prognosis of PAAD, as patients with high ptDNA levels have a better disease-free survival and OS.

In the current study, we tried to illustrate the prognostic value of eight CDCAs in PAAD patients. First, we compared the expression data and found that the eight CDCAs were increased in tumor tissues compared with normal tissues in most cancers and that the CDCAs act as risk factors of tumor OS in most cancers. For PAAD, increased CDCAs were observed in tumor tissues compared to normal tissues and were also observed in the advanced stage and grade PAADs. Increased risk of high CDCA expression was associated with poor prognosis, except for CDCA7 (non-significant) and CBX2 (opposite result). The prognostic value of CDCAs was also validated through a cell line experiment. Hu et al.^38^ found that the increased NUF2 expression in PAAD and determined that NUF2 could alter the proliferation and apoptosis of PAAD cell lines through LncRNA-AF339813. Zou et al. 39 revealed that CDCA3 expression was increased in the PAAD cell lines compared to normal human pancreatic duct epithelial cells, suggesting that knocking down CDCA3 can inhibit cell proliferation and promote cell apoptosis. Wang et al.^40^ revealed increased CDCA2 expression in PAAD tumors, and univariate analysis showed that increased CDCA2 expression is a risk factor for PAAD patients. Based on the TCGA-PAAD and ICGC-PACA cohorts, we determined that the integrative risk score based on the expression of eight CDCAs is a good predictor of the prognosis of PAAD patients.

The multicorrelated genes of the eight CDCAs were revealed in a Venn diagram. We found that the CDCAs not only impacted the biological process of the cell cycle but were also involved in DNA replication and repair-associated pathways. With the help of GSVA and GSEA, the impact of CDCAs on the cell cycle and DNA repair pathways was confirmed again, and the E2F, P53, and PI3K signaling pathways were also identified. CDCA2 could recruit the protein phosphatase 1 to chromatin, which impacted the antagonist function of ataxia telangiectasia mutated (ATM)-related signal transduction. DNA damage is fully impacted by the role of ATM kinase; the cascade of ATM kinase phosphorylation can inhibit p53-MDM2 interaction, ultimately leading to p21-induced G1 cell cycle arrest^41^. CDCA4 is a TRIP-Br transcriptional co-factor and can regulate the transcriptional activities of P53 and E2F1 transcription factors and impact the transcriptional regulation and cell fate determination through JUN oncogenes^42^. CBX2 is overexpressed in breast cancer and plays an essential role in tumor progression through the PI3K/AKT pathway^43^.

## Conclusion

CDCAs can predict the OS prognosis of PAAD patients. The cell cycle, DNA repair, E2F, P53 and PI3K signaling pathways, in which CDCAs are involved, impact the tumorigenesis of PAAD.

## Figures and Tables

**Table 1:** The prognostic values of CpG sites in the *CDCAs* by MethSurv.

CpG site	Gene symbol	Group	CpG Island	HR	CI	P value
cg21305265	CDCA2	TSS1500;1stExon;5'UTR	Island	0.535	0.346-0.828	0.005
cg07446572	CDCA3	Body	N_Shore	0.469	0.277-0.795	0.005
cg15942562	CDCA3	Body	N_Shore	0.603	0.385-0.945	0.027
cg25700897	CDCA3	3'UTR	N_Shelf	1.787	1.16-2.754	0.009
cg09936907	CDCA3	TSS200;TSS1500	Island	1.622	1.086-2.422	0.018
cg10095089	CDCA3	TSS1500;TSS200	N_Shore	1.67	1.031-2.704	0.037
cg10124440	CDCA3	TSS1500;5'UTR	N_Shore	0.51	0.298-0.873	0.014
cg02379052	CDCA4	5'UTR	Island	0.469	0.277-0.794	0.005
cg00047844	CBX2	Body	Island	1.512	1.004-2.276	0.048
cg09821032	CBX2	TSS1500	N_Shore	0.502	0.308-0.818	0.006
cg15209885	CBX2	Body	S_Shore	0.537	0.314-0.919	0.023
cg17346145	CBX2	Body	N_Shelf	0.607	0.373-0.985	0.043
cg18045515	CBX2	TSS1500	N_Shore	0.598	0.369-0.97	0.037
cg21848700	CBX2	TSS1500	Island	1.645	1.01-2.678	0.045
cg27347140	CBX2	TSS1500	N_Shore	0.505	0.302-0.845	0.009
cg05428978	CDCA7	Body	Island	0.637	0.408-0.995	0.048
cg05756320	CDCA7	Body	Open_Sea	1.75	1.061-2.888	0.028
cg07302848	CDCA7	TSS200	N_Shore	0.558	0.336-0.927	0.024
cg13484295	CDCA7	Body	Island	0.572	0.346-0.946	0.03
cg13854747	CDCA7	Body	Open_Sea	1.549	1.034-2.32	0.034
cg14906304	CDCA7	Body	Open_Sea	1.661	1.005-2.747	0.048
cg17336638	CDCA7	Body	Open_Sea	1.674	1.022-2.742	0.041
cg21583565	CDCA7	TSS1500	N_Shore	0.54	0.326-0.896	0.017
cg24937696	CDCA7	TSS200	Island	0.59	0.357-0.976	0.04
cg12751733	CDCA8	TSS1500	Island	1.574	1.005-2.466	0.047
cg27171474	CDCA8	Body	S_Shelf	1.58	1.027-2.431	0.038

**Table 2:** Upregulated gene sets in CDCAs determined high-risk phenotype by GSEA.

NAME	NES	P value
KEGG_PENTOSE_PHOSPHATE_PATHWAY	1.905	<0.001
KEGG_HOMOLOGOUS_RECOMBINATION	1.749	<0.001
KEGG_CELL_CYCLE	1.832	0.002
KEGG_OOCYTE_MEIOSIS	1.765	0.004
KEGG_P53_SIGNALING_PATHWAY	1.882	0.004
KEGG_PROTEASOME	1.826	0.004
KEGG_MISMATCH_REPAIR	1.73	0.004
KEGG_PORPHYRIN_AND_CHLOROPHYLL_METABOLISM	1.797	0.006
KEGG_DNA_REPLICATION	1.791	0.006
KEGG_GALACTOSE_METABOLISM	1.701	0.006
KEGG_PROGESTERONE_MEDIATED_OOCYTE_MATURATION	1.596	0.008
KEGG_PYRIMIDINE_METABOLISM	1.71	0.008
KEGG_THYROID_CANCER	1.675	0.008
KEGG_ONE_CARBON_POOL_BY_FOLATE	1.681	0.008
KEGG_ENDOCYTOSIS	1.609	0.008
KEGG_PATHOGENIC_ESCHERICHIA_COLI_INFECTION	1.749	0.008
KEGG_AXON_GUIDANCE	1.645	0.01
KEGG_AMYOTROPHIC_LATERAL_SCLEROSIS_ALS	1.639	0.01
KEGG_GLYCOLYSIS_GLUCONEOGENESIS	1.733	0.014
KEGG_STARCH_AND_SUCROSE_METABOLISM	1.63	0.015
KEGG_APOPTOSIS	1.612	0.016
KEGG_BASE_EXCISION_REPAIR	1.704	0.018
KEGG_DRUG_METABOLISM_OTHER_ENZYMES	1.684	0.018
KEGG_NUCLEOTIDE_EXCISION_REPAIR	1.611	0.02
KEGG_PURINE_METABOLISM	1.486	0.026
KEGG_PANCREATIC_CANCER	1.572	0.032
KEGG_SPLICEOSOME	1.57	0.034
KEGG_RNA_DEGRADATION	1.511	0.036
KEGG_ADHERENS_JUNCTION	1.573	0.044
KEGG_REGULATION_OF_ACTIN_CYTOSKELETON	1.503	0.046
KEGG_RIG_I_LIKE_RECEPTOR_SIGNALING_PATHWAY	1.531	0.047
